# Biofertilizer: The Future of Food Security and Food Safety

**DOI:** 10.3390/microorganisms10061220

**Published:** 2022-06-14

**Authors:** Augustine Innalegwu Daniel, Adewale Oluwaseun Fadaka, Arun Gokul, Olalekan Olanrewaju Bakare, Omolola Aina, Stacey Fisher, Adam Frank Burt, Vuyo Mavumengwana, Marshall Keyster, Ashwil Klein

**Affiliations:** 1 Plant Omics Laboratory, Department of Biotechnology, University of the Western Cape, Robert Sobukwe Road, Bellville 7530, South Africa; 4177137@myuwc.ac.za (O.A.); 3266314@myuwc.ac.za (S.F.); 2 Department of Biochemistry, Federal University of Technology, P.M.B 65, Minna 920101, Niger State, Nigeria; 3 Department of Science and Innovation/Mintek Nanotechnology Innovation Centre, Biolabels Node, Department of Biotechnology, University of the Western Cape, Robert Sobukwe Road, Bellville 7530, South Africa; afadaka@uwc.ac.za (A.O.F.); vuyom@sun.ac.za (V.M.); 4 Department of Plant Sciences, Qwaqwa Campus, University of the Free State, Phuthadithjaba 9866, South Africa; gokula@ufs.ac.za; 5 Environmental Biotechnology Laboratory, Department of Biotechnology, University of the Western Cape, Robert Sobukwe Road, Bellville 7530, South Africa; 3779970@myuwc.ac.za (O.O.B.); 3238864@myuwc.ac.za (A.F.B.); mkeyster@uwc.ac.za (M.K.); 6 Division of Molecular Biology and Human Genetics, Faculty of Medicine and Health Sciences, DST-NRF Centre of Excellence for Biomedical Tuberculosis Research, South African Medical Research Council Centre for Tuberculosis Research, Stellenbosch University, Cape Town 7505, South Africa

**Keywords:** biofertilizers, ecofriendly, food safety, food security, growth hormones, nitrogen fixation

## Abstract

There is a direct correlation between population growth and food demand. As the global population continues to rise, there is a need to scale up food production to meet the food demand of the population. In addition, the arable land over time has lost its naturally endowed nutrients. Hence, alternative measures such as fertilizers, pesticides, and herbicides are used to fortify the soil and scale up the production rate. As efforts are being made to meet this food demand and ensure food security, it is equally important to ensure food safety for consumption. Food safety measures need to be put in place throughout the food production chain lines. One of the fundamental measures is the use of biofertilizers or plant growth promoters instead of chemical or synthesized fertilizers, pesticides, and herbicides that poise several dangers to human and animal health. Biofertilizers competitively colonize plant root systems, which, in turn, enhance nutrient uptake, increase productivity and crop yield, improve plants’ tolerance to stress and their resistance to pathogens, and improve plant growth through mechanisms such as the mobilization of essential elements, nutrients, and plant growth hormones. Biofertilizers are cost-effective and ecofriendly in nature, and their continuous usage enhances soil fertility. They also increase crop yield by up to about 10–40% by increasing protein contents, essential amino acids, and vitamins, and by nitrogen fixation. This review therefore highlighted different types of biofertilizers and the mechanisms by which they elicit their function to enhance crop yield to meet food demand. In addition, the review also addressed the role of microorganisms in promoting plant growth and the various organisms that are beneficial for enhancing plant growth.

## 1. Introduction

Global demand for agricultural products is increasing due to the increasing human population [1]. There are already about 7.9 billion people on the planet, and this number is expected to rise, with a projected growth of almost 10 billion in the next 50 years [2,3,4]. As the world’s population continues to increase, so does the demand for food; hence, feeding the current vast population, which will certainly grow with time, is a significant task [5]. To meet the challenges of food scarcity caused by the rise in population, various agricultural alternatives such as the use of chemical or synthetic fertilizers, pesticides, and insecticides have been used to produce crops with high yield within the shortest time possible and to protect them from insects and pest attack during and after harvest [6]. However, the use of these fertilizers and insecticides has raised much public concern about the sustainability, safety, and security of the food supply [7,8]. Studies have shown that there is a significant amount of pesticide residue present in foodstuffs long after they are taken away from farms for human consumption [9]; hence, the need for alternatives such as biofertilizer in ensuring food safety and security [6]. Moreover, synthetic fertilizers that consist of various nutrients such as nitrogen (N_2_), phosphorus (P), potassium (K), and sulfur may become harmful if used beyond the required amount [2]. The harmful effects of these fertilizers include the weakening of plant roots, the high rate of disease incidence, soil acidification [10], and eutrophication of ground water and other water bodies [11]. Nutrients such as nitrates leach to groundwater and cause “blue baby syndrome”, also called “acquired methemoglobinemia” [2]. The impact of these chemicals will not only affect the present but also future generations. Therefore, there is need to search for eco-friendly approaches such as biofertilizers, which play a major role in sustainable agriculture [2].

Biofertilizers are microorganisms that support the growth of plants by enhancing the nutrient supply to the host plant when given to seeds, plants, or the soil [2,12,13]. They colonize the rhizosphere or the inside of the plants. This entails the use of plant growth-promoting microorganisms that participate in a variety of biotic activities in the soil ecosystem in order to make it dynamic and sustainable for crop development. Biofertilizers are widely used to accelerate microbial activities that increase the availability of nutrients that plants can easily absorb. They increase soil fertility by fixing atmospheric N_2_ and solubilizing insoluble phosphates in the soil, resulting in plant growth-promoting chemicals [14]. These biofertilizers make use of the naturally available biological system of nutrient mobilization, which greatly enhances soil fertility and, as a result, crop productivity. [14]. It has been reported that the biofertilizer market is estimated to grow at a compound annual growth rate (CAGR) of 14.0% from 2015 to 2020 and is expected to reach USD 1.88 billion by 2025 [15]. Because of strict regulations on the use of chemical fertilizers, biofertilizers are the most widely used in Europe and Latin America [15].

The words “food security” and “food insecurity” are commonly used in discussions of global conditions and prospects. Food security is defined as the availability and accessibility of safe and nutritious food that fits the dietary requirements of a healthy and active lifestyle. Food insecurity occurs when people do not have enough access to safe and nutritious food, and thus do not consume enough to live an active and healthy life. This could be due to a lack of food, a lack of purchasing power, or inefficient use of resources at the household level [16]. Another factor that may be responsible for food insecurity may be the depletion of soil nutrients resulting from continuous tillage and the use of chemical or synthetic fertilizers for continuous agricultural production. This have made the soil lose its fertility, and most of the agricultural produce consumed is not safe because of the chemical residues that are left in them. This review highlights the role of biofertilizers in crop improvement and the production of safe and secure food, the mechanisms of microorganisms in enhancing plant growth, and the various types of organisms used as plant growth-promoting microorganisms.

## 2. Biofertilizers

Microbial inoculants, also known as biofertilizers, are organic products that contain specific microorganisms obtained from plant roots and root zones. They have been found to boost plants’ growth and yield by 10–40% [16]. These bioinoculants colonize the environment when applied to the rhizosphere and the interior of the plant to promote plant growth [17]. They not only add nutrients to the soil to improve soil fertility and crop yield, but they also protect the plant against pests and diseases. They have been shown to enhance seedling survival, extend the root system’s life, eliminate harmful chemicals, and shorten flowering time [11]. Another advantage is that biofertilizers are no longer necessary after 3–4 years of continuous use, since the parental inocula are sufficient for growth and multiplication [18]. Plants require 17 essential elements for effective growth and development. N_2_, P, and K are all required in significant amounts [18]. Several microorganisms, including nitrogen-fixing soil bacteria and cyanobacteria, phosphate-solubilizing bacteria, molds, and mushrooms, are routinely utilized as biofertilizers [19]. Similarly, microorganisms that produce phytohormones are used in the production of biofertilizers. They feed the plant with growth-promoting compounds such as indole acetic acid (IAA), amino acids, and vitamins, as well as improving the soil’s productivity and fertility while conserving crop yield [20].

### 2.1. Types of Biofertilizers

Biofertilizers are divided into groups based on their functions and mechanisms of action. The most commonly used biofertilizers are nitrogen-fixers (N-fixers), potassium solubilizers (K solubilizers), phosphorus solubilizers (P solubilizer), and plant growth-promoting rhizobacteria (PGPR) [17]. One gram of rich soil can contain up to 10^10^ cfu bacteria, with a live weight of 2000 kg/ha [21]. Cocci (spheres with a diameter of 0.5 m), bacilli (rods with a diameter of 0.5–0.3 m), and spirals with a diameter of 1–100 m are all types of soil bacteria. The frequency of bacteria in the soil is influenced by the physical and chemical properties of the soil, organic matter, and phosphorus concentration, as well as cultural activities. Nutrient fixation and improvements in plant growth by bacteria, on the other hand, are critical components for accomplishing future sustainable agricultural goals. Microbes also help the ecosystem’s numerous nutrient cycles. Table 1 summarizes the classification of biofertilizers based on the type of microbe utilized and the mechanism of action, as well as appropriate examples.

### 2.2. The Role of Plant Growth-Promoting Microorganisms in Crop Production

Plants are exposed to diverse microorganisms in their natural habitat, including bacteria, fungi, algae, and protozoa. The majority of these microorganisms occur in the soil’s rhizosphere in various types of association, some as free-living organisms, while others associate with plant roots or even live within root or shoot tissues as endophytes [26,27]. In the instance of a symbiotic relationship with nitrogen-fixing bacteria in the root nodules of leguminous plants, these connections may be advantageous to the plant, while others may be parasitic, pathogenic, or have no known effect on plant growth or development [27]. Microorganisms that promote plant growth are involved in a variety of biotic activities in the soil ecosystem to keep it dynamic and sustainable for crop production [28]. They colonize plant roots competitively and improve plant growth through a variety of mechanisms, including phosphate solubilization [29]; nitrogen fixation [30]; production of indole-3-acetic acid (IAA), siderophores [31], 1-amino-cyclopropane-1-carboxylate (ACC) deaminase, and hydrogen cyanate [32]; degradation of environmental pollutants; and the production of hormones, antibiotics, and lytic enzymes [33]. Furthermore, some plant growth-promoting rhizobacteria may be able to stimulate additional particular plant growth-promoting properties, such as heavy metal detoxification, salinity tolerance, and biological control of phytopathogens and insects [34].

*Desulfovibrio*, *Rhodospirillum*, and *Rhodopseudomonas* are examples of beneficial microbes that create symbiotic partnerships with plants, exchanging carbon-based photo-assimilates for minerals ingested by the microbe. Plant and soil biologists have paid extensive attention to beneficial symbiotic microorganisms in recent years, with major goals being the identification and adoption of new, environmentally beneficial lines of plant growth-promoting (PGP) microorganisms. Plant growth stimulators have also been found in other biostimulators, such as those found in seaweed extracts or decomposed vegetation [26].

## 3. Mechanisms of Action of Plant Growth-Promoting Rhizobia

**Figure 1:**
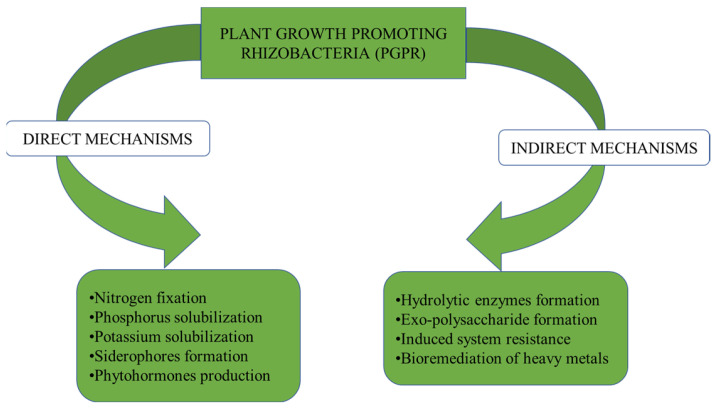
Mechanisms of plant growth-promoting rhizobia.

There are different mechanisms by which plant growth-promoting rhizobacteria stimulate the growth of plants. They are widely classified as direct or indirect mechanisms [35]. Moreover, depending on their association with the plants, plant growth-promoting rhizobacteria are grouped as both symbiotic bacteria and free-living rhizobacteria [35]. Examples of plant growth-promoting bacteria include the free-living bacteria which form distinct symbiotic relationships with plants, endophytic bacteria which colonize some portions of plant tissue, and cyanobacteria [35]. Despite the differences that exist among the bacteria, they all show a similar type of mechanism while promoting bacterial growth [35]. The bacteria may use one of two methods to promote plant growth by (i) directly by improving resource acquisition or changing the plant’s hormone levels, or (ii) indirectly by lowering the inhibitory effects of various pathogenic agents on plant growth and development (Figure 1).

### 3.1. Direct Mechanisms

#### 3.1.1. Facilitating Resource Acquisition

Biofertilizer aids in nitrogen fixation, iron sequestration, and phosphate solubilization, allowing plants to use these complex organic molecules.

#### 3.1.2. Nitrogen Fixation

One of the most important nutrients for plant growth is nitrogen. Although our atmosphere contains around 80% gaseous nitrogen, green plants are unable to utilize it directly [36]. Biological nitrogen fixation is the conversion of atmospheric nitrogen to ammonia by soil-borne microbes. About 175 × 10^6^ tons of nitrogen are fixed globally each year by nitrogen-fixing bacteria [37]. Biological nitrogen fixation is a critical component of microbial activities. Only prokaryotes, which can be symbiotic or free-living in nature, are able to produce the nitrogenase enzyme to fix nitrogen biologically. The enzyme nitrogenase catalyzes biological nitrogen fixation. Some soil bacteria and blue-green algae can convert nitrogen from the air into ammonia in their cells. Diazotrophy, or nitrogen fixation, is the process of nitrogen reduction [29,37,38,39,40]. N-fixers, also known as diazotrophs, are microbes that reduce atmospheric nitrogen. Plants can directly utilize the ammonia produced during nitrogen fixation.

#### 3.1.3. Rhizobacteria

**Figure 2:**
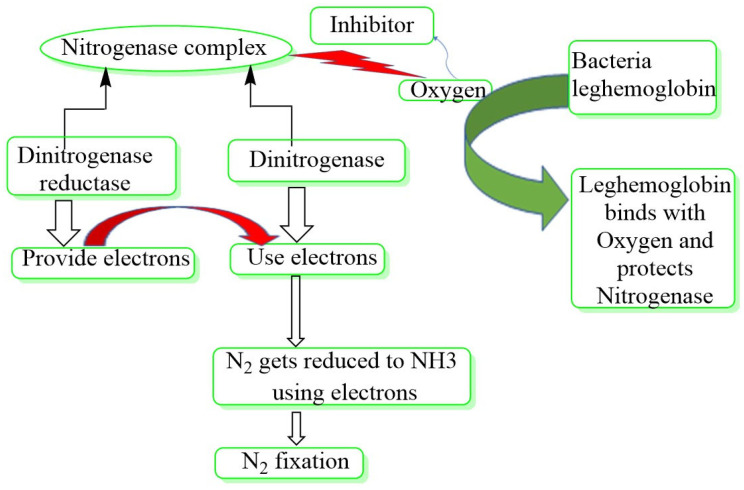
Plant growth-promoting rhizobacteria’s molecular N_2_ fixing mechanism. The nitrogen fixation process is carried out by the nitrogenase enzyme complex, which comprises dinitrogenase reductase and dinitrogenase. Dinitrogenase reductase produces electrons, which dinitrogenase uses to convert N_2_ to NH_3_. Because the enzyme complex can attach to O_2_ and become inactive, oxygen is a powerful inhibitor. Bacterial leghemoglobin, on the other hand, has a higher affinity for oxygen and hence binds to free oxygen more effectively. As a result, the presence of leghemoglobin protects the nitrogenase enzyme complex from oxygen, keeping it active [16].

The *Rhizobiaceae* (α-proteobacteria) are a family of symbiotic N_2_-fixing rhizobacteria that live in a symbiotic association with leguminous plant roots. This relationship necessitates a complicated interaction between the host and the symbiont, which leads to the creation of nodules that house the rhizobia as an intracellular symbiont [41]. The rhizobia include *Rhizobium*, *Bradyrhizobium*, *Sinorhizobium*, *Azorhizobium*, and *Mesorhizobium* as a group. Rhizobacteria that fix nitrogen in non-leguminous plants are known as non-symbiotic rhizobacteria. They are also known as diazotrophs, and they can create a non-obligate relationship with their hosts [42]. The nitrogen fixation process is carried out by nitrogenase, a complex enzyme structure that includes dinitrogenase reductase, which has iron (Fe) as a cofactor, and dinitrogenase, which has iron (Fe) and molybdenum (Mo) as cofactors [39]. In Figure 2, dinitrogenase reductase produces electrons and uses them to decrease N_2_ to NH_3_ [43]. Mo-nitrogenase, V-nitrogenase, and Fe-nitrogenase are three different nitrogenase complexes based on changes in the cofactor of dinitrogenase [40,44,45]. N_2_ fixation genes, also known as *Nif* genes, are found in both symbiotic and free-living nitrogen-fixing microorganisms [44]. *Nif* genes are structural genes involved in Fe–protein activation, Fe–Mo cofactor biosynthesis, electron donation, and serve as regulatory genes required for enzymatic synthesis and activity [45]. Despite being a negative regulator of *Nif* gene expression, oxygen is required for *Rhizobium* sp. bacteroid respiration [46]. Because bacterial leghemoglobin has a high affinity for oxygen, it can keep the enzyme active even in the absence of oxygen (Figure 2). To efficiently pursue the nitrogen fixation process, sufficient O_2_ supply to the bacteroid for respiration must occur concurrently with prevention of the O_2_ supply to the nitrogenase enzyme complex. The simplest way to accomplish this objective is to use genetic engineering to introduce bacterial hemoglobin (Hb) that binds O_2_ to the rhizobacteria [47]. Following this strategy, it was discovered that after transforming *Rhizobium etli* with the Hb gene of *Vitreoscilla* sp. (a Gram-negative bacterium), the rhizobial cells had a two- to threefold faster respiration rate than non-transformed rhizobial cells [30]. Because *Vitreoscilla* sp. has Hb-producing genes, inserting this gene into rhizobial cells resulted in Hb production in the transformed cells. Despite the low availability of O_2_, the Hb generated in this way could bind to it with a high affinity. When the altered *Rhizobium* was inoculated to bean plants, the plants had 68% greater nitrogenase activity than plants inoculated with wild-type *R. etli*. The resulting seeds had a 25–30% increase in leaf content and a 16% rise in nitrogen content as a result of this change [48]. 

Nodule development is another key component of *Rhizobium*. To accommodate the symbiotic bacteria *Rhizobium*, most bean plants generate root lateral organs de novo, known as “root nodules”. Symbiotic bacterial infection of the legume plant stimulates the creation of new organs, such as nodules, by altering the fate of differentiated cortical cells [49]. To establish optimal nodule development, two regulatory events, bacterial infection and nodule organogenesis, must be coordinated in the epidermis and cortical cells, respectively, during this process [50]. The symbiotic reactions between the host legume plants and *Rhizobium* are sustained by nodulation factors (Nod factors), which are lipochitin oligosaccharides released by rhizobia [51]. Plant ethylene levels were found to be higher after *Rhizobium* sp. infection of legumes, and this higher ethylene concentration inhibited further rhizobial infection and nodule development [16]. By producing a small compound molecule called “rhizobitoxine”, some rhizobial strains can enhance the number of nodules formed on the host bean plant’s roots by restricting the rise in ethylene production [52].

Rhizobitoxine is a phytotoxin that inhibits ethylene biosynthesis by chemically inhibiting the enzyme 1-aminocyclopropane-1-carboxylate (ACC) synthase [53]. ACC deaminase is an enzyme produced by some rhizobial strains that eliminates some of the ACC (the immediate precursor to ethylene in plants) before it is converted to ethylene. The plant’s nodule production and biomass increase by 25–40% as a result of this reduction [54]. Because around 1–10% of rhizobial strains in the field naturally contain ACC deaminase, it is possible to improve the nodulation effectiveness of rhizobia strains without ACC deaminase by genetically engineering them with rhizobia ACC deaminase genes [3]. The introduction of an ACC deaminase gene from *Rhizobium leguminosarum* bv. *viciae* into the chromosomal DNA of a *Sinorhizobium meliloti* strain that lacked this enzyme enhanced nodule numbers by 35% and host alfalfa plant biomass by 40% compared with the wild-type control strain [30,48]. *Azorhizobium* is a stem nodule-forming symbiotic bacterium that forms stem nodules and fixes N_2_, among other *Rhizobium* strains [55]. They also make a large amount of indole acetic acid (IAA), which helps plants thrive. *Bradyrhizobium* is a good nitrogen fixer, and when it was inoculated into *Mucuna* seeds, it boosted total organic carbon, N_2_, P, and K levels in the soil. As a result, it boosted plant growth, soil microbial population, and plant biomass and lowered the weed population [11].

#### 3.1.4. Azospirillum

*Azospirillum* is a Gram-negative, aerobic nitrogen-fixing bacteria that do not form nodules and belong to the Spirilaceae family [56]. Although there are several species in this genus, such as *Azospirillum amazonense*, *Azospirillum halopraeferens*, and *Azospirillum brasilense*, *Azospirillum lipoferum* and *A. brasilense* are the most beneficial [57]. Because they develop and fix nitrogen on the organic salts of malic and aspartic acid, *Azospirillum* forms associative symbiosis with many plants, notably those with the C_4_ dicarboxylic pathway (Hatch–Slack pathway) of photosynthesis [58]. As a result, it is mostly suggested for maize, sugarcane, sorghum, pearl millet, and other crops. They make growth stimulants (IAA, gibberellins, and cytokinin) that help in root development and nutrient uptake (N, P, and K). Inoculation with *Azospirillum* has a significant impact on root development and exudation [59]. When *A. brasilense* sp. 245 was inoculated to maize, the production of various phytohormones increased noticeably, resulting in a significant increase in maize growth [16]. The root physiology and architecture of maize were altered as a result of the increased synthesis of several phytohormones, resulting in an increase in mineral intake by the plant [16]. Inoculation with *Azospirillum* and *Pseudomonas* altered the cultivable bacterial community in the wheat rhizosphere, according to Naiman et al. [60]. They also found that inoculating the soil microflora with *Azospirillum* and *Pseudomonas* altered the profiles of carbon source use during the tillering and grain filling stages [60]. Inoculation with two *A. brasilense* strains (40 and 42 M) isolated from maize roots was also found to affect the community-level physiological profiles of the cultivable microbial communities associated with rice [59].

#### 3.1.5. Azotobacter

*Azotobacter* is a genus of non-symbiotic, free-living, aerobic, photoautotrophic bacteria belonging to the *Azotobacteriaceae* family. *Azotobacter chroococcum* is the most frequent species in arable soils [61]. They are usually found in neutral and alkaline soils. *Azotobacter vinelandii*, *Azotobacter beijerinckii*, *Azotobacter insignis*, and *Azotobacter macrocytogenes* are among the other species identified [57]. They produce the Vitamin B complex and various phytohormones such as gibberellins, naphthalene acetic acid (NAA), and other compounds that prevent root infections while promoting root growth and mineral uptake [62]. *Azotobacter* has been found to release chemicals that limit the growth of certain root infections while also improving root growth and nutrient uptake [16]. *Azotobacter* has also been found to add 15–93 kg N/ha to *Paspalum notatum* roots [11]. Another strain, *Azotobacter indicum*, can produce a variety of antifungal antibiotics that are utilized to reduce seedling mortality by inhibiting the growth of many harmful fungi in the root region [63]. *Azotobacter* populations are often low in the rhizosphere of crop plants and in uncultivated soils, according to research. This organism has been found in the rhizosphere of a variety of crops, including rice, maize, sugarcane, bajra, vegetables, and plantation crops [64].

#### 3.1.6. Blue-Green Algae (Cyanobacteria)

The blue-green algae are photosynthetic organisms that belongs to eight different families. They promote plant growth by generating auxin, indole acetic acid, and gibberllic acid, as well as fixing roughly 20–30 kg N/ha in submerged rice fields [57]. For lowland rice production, nitrogen is one of the main nutrients required in high quantities. Soil nitrogen and biological nitrogen fixation (BNF) by related microorganisms are the two main sources of nitrogen [43,65]. Fungi, liverworts, ferns, and flowering plants create symbiotic relationships with blue-green algae [45]. *Anabena oryzae*, *Nostoc calcicola*, and *Spirulina* sp. are three blue-green algae that have been shown to reduce the quantity of galls and egg masses induced by the root-knot nematode *Meloidogyne incognita* infecting cowpea, and to improve plant growth [16].

#### 3.1.7. Azolla

*Azolla* has a 4–5% nitrogen content on a dry basis and 0.2–0.4% on a wet basis. In rice production, it can be a valuable source of organic manure and nitrogen [57]. The important aspect of using *Azolla* as a biofertilizer is that it decomposes quickly in the soil and provides nitrogen to rice plants efficiently. In addition, it adds to the provision of phosphorus, potassium, zinc, iron, molybdenum, and other micronutrients [66]. Prior to rice cultivation, *Azolla* can be utilized as a green biofertilizer in the fields. *Azolla pinnata* is the most commonly used species in India, and it may be produced commercially through vegetative techniques [14]. *Azolla caroliniana*, *Azolla microphylla*, *Azolla filiculoides*, and *Azolla mexicana* are some of the other *Azolla* species that have been introduced to India for their huge biomass output [57].

#### 3.1.8. Phosphate Solubilization

Despite the fact that phosphorus is abundant in the soil, the majority of it is insoluble and hence is inaccessible to support plant growth, since plants only absorb it in two soluble forms: monobasic and dibasic. Inorganic phosphorus, such as apatite, or organic phosphorus, such as inositol phosphate (soil phytate), phosphomonoesters, and phosphotriesters, may be present [67]. Furthermore, much of the soluble inorganic phosphorus used in chemical fertilizers is quickly immobilized after being applied to the field. As a result, it is unavailable to plants and hence is wasted [67]. This has prompted researchers to look for environmentally benign and cost-effective ways to boost crop output in low-phosphorus soils. Microbes that can solubilize inorganic phosphorus play a critical role in these settings as a potential option for providing phosphorus to the plants. As a result, they are regarded as a promising biofertilizer, since they may provide the necessary phosphorus to plants, even from low-quality sources [14].

**Figure 3:**
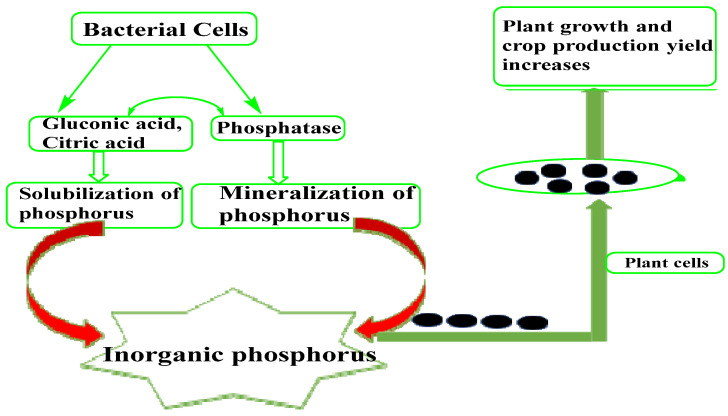
Phosphate-solubilizing rhizobacteria solubilize inorganic phosphorus. Inorganic phosphorus is solubilized by bacteria using organic acids with a low molecular weight such as gluconic and citric acids. These acids’ hydroxyl (OH) and carboxyl (COOH) groups chelate the phosphate-bound cations, converting insoluble phosphorus into a soluble organic form. Mineralization of soluble phosphorus is accomplished through the production of several phosphatases, which catalyze the hydrolysis process. When plants absorb these solubilized and mineralized phosphorus molecules, their overall growth and crop output improve dramatically [11].

Organic acids with a low molecular weight such as gluconic and citric acids, which are generated by several soil microorganisms, are responsible for inorganic phosphorus solubilization [30]. Figure 3 depicts a schematic diagram of phosphate solubilization by microorganisms. The hydroxyl and carboxyl groups in these low-molecular-weight organic acids can chelate the cations attached to phosphate, resulting in the conversion of insoluble phosphorous to its soluble form. The mineralization of organic phosphorus, on the other hand, is accomplished by the production of several phosphatases that catalyze the hydrolysis of phosphoric esters [68]. Above all, phosphate solubilization and mineralization can occur in the same bacterial strain [69]. *Pseudomonas*, *Bacillus*, *Rhizobium*, *Burkholderia*, *Achromobacter*, *Agrobacterium*, *Micrococcus*, *Acetobacter*, *Flavobacterium*, and *Erwinia* are among the bacteria that have the ability to solubilize insoluble inorganic phosphorus [11]. Phosphate-solubilizing bacteria are commonly found in large numbers in soils and plant rhizospheres. These comprise aerobic and anaerobic strains, with aerobic strains being more common in submerged soils [69]. However, it has been discovered that the rhizosphere has a larger concentration of phosphate-solubilizing bacteria (PSB) than non-rhizosphere soil [11]. PSB stimulate the efficacy of biological nitrogen fixation (BNF) by nitrogen-fixing bacteria, in addition to delivering phosphorus in soluble form to plants [70].

#### 3.1.9. Sequestering Iron

**Figure 4:**
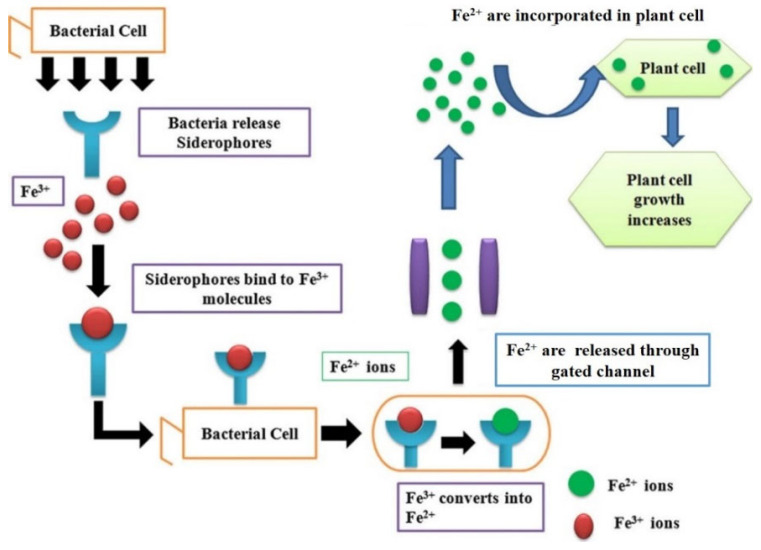
Plant growth-promoting rhizobacteria produce siderophores, which are used to sequester iron. Bacteria release low-molecular-weight iron chelators known as “siderophores,” which have high affinity for Fe^3+^, bind firmly to it, and are taken up by bacteria. Fe^3+^ is converted to Fe^2+^ inside the bacterial membrane, and Fe^2+^ is discharged into the cell via a gated channel that connects the bacteria’s inner and outer membranes. The total plant growth improves significantly when the host plant integrates these soluble Fe^2+^ molecules produced by the bacteria.

Iron is an essential ingredient for practically all living things. Iron is required by all plants, animals, and microbes [16]. Iron exists as Fe^3+^ in an aerobic environment and is prone to generating insoluble hydroxides and oxyhydroxides. As a result, the majority of iron is unavailable for absorption by bacteria and plants [16]. In general, bacteria obtain iron via secreting siderophores, which are low-molecular-weight iron chelators with a high affinity for complex iron (Figure 4). The majority of siderophores are water-soluble, and they are classified as extracellular or intracellular siderophores [16]. *Rhizobacteria* differ in their ability to use siderophore cross-linking. Some *Rhizobacteria* use homologous siderophores proficiently, while others use heterologous siderophores [45,71]. Iron is reduced from Fe^3+^ to Fe^2+^ in the bacterial membrane in both Gram-positive and Gram-negative bacteria, and then released into the cell through siderophores via a gating mechanism that connects the inner and outside membranes (Figure 4). Under iron-limiting conditions, siderophores operate as solubilizing agents for iron from minerals or organic molecules [72]. Similar to iron, siderophores create stable complexes with other heavy metals, as well as radioactive particles such as uranium and neptunium [73]. The concentration of soluble metal increases when the siderophores bind to a heavy metal. As a result, bacterial siderophores assist the host plant to reduce the stress caused by elevated heavy metal levels in the soil [30]. Plants absorb iron from bacterial siderophores using a variety of processes, including chelation and release, direct uptake of siderophore–Fe complexes, and ligand exchange reactions [74]. According to Thomine and Lanquar [74], siderophores facilitated iron transfer in oat plants and elevated plant growth. Rhizophore-produced siderophores delivered iron to the oat plant, which possesses a mechanism for utilizing Fe siderophores when iron is scarce [74]. *Pseudomonas fluorescens* C_7_ generated the Fe–pyoverdine complex, which was taken up by *Arabidopsis thaliana* plants, resulting in a rise in iron levels in plant tissues and improved plant growth [71]. When plants are exposed to stress situations such as heavy metal pollution, the availability of iron to plants by soil bacteria becomes extremely important. In this case, siderophores can also assist plants to cope with the stress caused by high amounts of heavy metals [73].

#### 3.1.10. Modulation of Phytohormone Levels

Plant hormones, also known as phytohormones, play a crucial role in plant growth and development [16]. When plants are exposed to growth-limiting environmental conditions, evidence suggests that they modify their endogenous phytohormone levels to reduce the detrimental impacts of environmental stress [16]. Microorganisms in the rhizosphere have been found to produce or modify phytohormone levels in the host plants. As a result, by modifying the level of endogenous phytohormones in the host plants, they can considerably influence the hormonal balance and stress response of the host plant [30]. For a long period, scientists have known that bacteria produce the phytohormone auxin (indole-3-acetic acid/indole acetic acid/IAA). According to one study, 80% of microorganisms isolated from the rhizosphere of diverse crops are capable of generating and releasing auxins as secondary metabolites [72].

Indole-3-acetic acid (IAA) is involved in many aspects of plant growth and development, as well as defense responses. The exceptional complexity of IAA biosynthesis, its transport mechanisms, and the different signaling pathways involved in IAA synthesis and transport reflects this diversity of roles [75]. In general, IAA stimulates seed and tuber germination; increases the rate of xylem and root development; controls vegetative growth processes; initiates lateral and adventitious root formation; mediates responses to light, gravity, and fluorescence; and affects photosynthesis and pigment formation, the biosynthesis of various metabolites, and stress resistance [28]. Because IAA is involved in various cell division and vascular bundle creation processes, it appears that a higher level of IAA in the host legume plants is required for nodule development [29]. Furthermore, bacterial IAA increases the root surface area and root length, allowing the plant to acquire soil nutrients more easily [29].

Furthermore, rhizobacterial IAA loosens plant cell walls, allowing for greater root exudation, which offers additional nutrients to sustain bacterial development [29]. As a result, rhizobacterial IAA has been identified as a crucial effector molecule in both disease and phytostimulation in plant–microbe interactions [29]. The amino acid tryptophan is an important component that influences IAA production levels. Tryptophan has been identified as the principal precursor of IAA and has been shown to have an important role in altering IAA biosynthesis levels [16]. Starting with tryptophan, at least five distinct processes for the synthesis of IAA have been reported, most of which are comparable with the mechanisms discovered in plants, but a few intermediates differ in each case [76]. The production of IAA via indole-3-pyruvic acid and indole-3-acetic aldehyde is the first pathway. The majority of bacteria, including *Rhizobium*, *Azospirillum*, *Erwinia herbicola*, *Klebsiella*, and others, use this pathway. The conversion of tryptophan to indole-3-acetic aldehyde is the second process, which may include an alternate pathway by which tryptamine is generated. *Pseudomonas* and *Azospirilla* use this route. The biosynthesis of IAA occurs via indole-3-acetamide in the third route. *Agrobacterium tumefaciens*, *Pseudomonas syringae*, and other phytopathogenic bacteria use this pathway. The conversion of tryptophan into indole-3-acetonitrile is the fourth step for IAA biosynthesis. Cyanobacteria have this mechanism. The last mechanism, which is more widespread in plants, Cyanobacteria, and *Azospirilla*, is the production of IAA via a tryptophan-independent pathway. Although bacterial IAA has been implicated in almost every aspect of plant growth and development, the acquisition of bacterial IAA may modify the endogenous pool of plant IAA. The degree of endogenous IAA in plants is critical in determining whether bacterial IAA stimulates or hinders plant growth in this respect. Endogenous IAA has been determined to be either ideal or sub-optimal for plant root development [30].

### 3.2. Indirect Mechanisms

Synthesizing multiple types of antibiotics is the most common way for plant growth-promoting bacteria (PGPB) to limit plant pathogen proliferation [77,78]. Many of the compounds have been thoroughly researched and some have even been marketed. The majority of commercialized rhizobacterial products function as bio-inoculants to combat plant diseases rather than to improve plant nutrition or reduce abiotic stressors [77]. Plant illnesses caused by pathogens such as *Fusarium* spp., *Pythium* spp., *Rhizoctonia* spp., and *Sclerotium* spp. have been reported to be treated by using biofertilizers such as *Trichoderma harzianum*, *P. fluoresecens*, and *Bacillus subtilis*, which boost plant growth and overall output. Hydrogen cyanide (HCN), phenazines, pyrrolnitrin, 2,4-diacetylphloroglucinol, pyoluteorin, viscosinamide, and tensin are among the antifungal metabolites produced by various *Rhizobacteria* [38]. It has also been observed that the contact between some *Rhizobacteria* and plant roots can protect the host plant from pathogenic fungi, bacteria, and viruses. Induced systemic resistance (ISR) is the term for this phenomenon [79]. Furthermore, ISR does not necessitate any direct interaction between the pathogens and the resistance-inducing PGPB [30].

Induced systemic resistance (ISR) is caused by jasmonate and ethylene signaling in the host plant, which acts as a defense mechanism against a range of plant pathogens [30]. Many individual bacterial components, such as lipopolysaccharides (LPS), flagella, siderophores, cyclic lipopeptides, 2,4-diacetylphloroglucinol, and homoserine lactones, as well as volatile compounds such as 2,3 butanediol and acetonin, have been reported to cause ISR in the host plant, allowing the host plant to combat a variety of plant pathogens [79]. Some biocontrol bacteria generate enzymes such as chitinases, cellulases, 1,3-gluconases, proteases, and lipases that can lyse a section of the cell wall of many pathogenic fungi such as *Botrytis cinerea*, *Sclerotium rolfsii*, *Fusarium oxysporum*, *Phytophthora* spp., *Rhizoctonia solani*, and *Pythium ultimum* [80,81]. Some PGPB strains produce siderophores, which operate as a biocontrol agent. In this approach, PGPB’s siderophores prevent pathogens from acquiring adequate iron, limiting their growth and proliferation [80]. Because the siderophores produced by PGPB have a higher affinity for iron than the pathogens, this technique is effective. As a result, the infections’ ability to utilize iron is diminished and they are unable to multiply in the rhizosphere [30]. Plants have been reported to synthesize ethylene in response to a range of stressors, including fungal phytopathogenic infections [82]. When plant cells become infected, ethylene causes a stress/senescence response in the plant, which results in the death of cells that are either infected or present near the fungal infection site [82]. As a result, increasing levels of ethylene build up, as well as the infection caused by plant pathogens, causing a large amount of the harm to the plant. Exogenous ethylene has also been shown to exacerbate the severity of fungal infections. As a result, lowering the ethylene response is one strategy to reduce the harm produced by phytopathogen infections of the host plants [3]. Ethylene inhibitors have been found to not only reduce the ethylene response level but also to diminish the severity of fungal infections. When the host plant is affected by pathogens, the enzyme ACC deaminase found in PGPB can adjust the ethylene level [16]. As a result, the most straightforward strategy to reduce ethylene levels is to apply PGPB harboring the ACC deaminase gene to the plants (usually the roots or seeds).

## 4. Benefits of Biofertilizers in Food Production

To meet the increased need for food, continuous and indiscriminate usage of synthetic or chemical fertilizers has unquestionably resulted in contamination and ecosystem modification [16]. Even so, the long-term impacts of using synthetic or chemical fertilizers lower soil fertility and have resulted in the production of disease-prone crops [83,84]. The amount of food produced today compared with the amount required to feed everyone in 2050 is drastically lower. By 2050, the world’s population will have swelled to about 10 billion people, with roughly 4.5 billion more mouths to feed than in 2022. People will consume more resource-intensive, animal-based diets as their wages rise. To feed the growing population with a deficit amount of available nutrients, the world certainly needs to encourage agricultural productivity in a sustainable and ecofriendly way. Hence, it is necessary to re-evaluate many of the existing agricultural approaches, which include the use of chemical fertilizers, pesticides, herbicides, fungicides, and insecticides [85]. In light of the harmful effects of chemical or synthetic fertilizers, biofertilizers are supposed may be a safe alternative to chemical inputs and minimize alteration of the ecosystem to a great extent. Biofertilizers are cost-effective and ecofriendly in nature, and their prolonged use enhances soil fertility substantially [16]. It has been found that using biofertilizers increases crop yield by 10–40% by increasing protein, vital amino acids, and vitamins, and nitrogen fixation [86]. Biofertilizers provide a number of advantages, including being a low-cost source of nutrients, excellent suppliers of micro-compounds and micronutrients, organic matter suppliers, growth hormone producers, and a means of counteracting the negative effects of chemical fertilizers [87]. Different microorganisms are important components of soil, and they play a key role in a variety of biotic activities in the soil ecosystem that keep the soil active for nutrient mobilization and long-term crop development [45].

## 5. Conclusions and Future Perspectives

The continuous rise in the global population has translated to a direct increase in the demand for food production. The use of these biofertilizers has been reported to boost the food production rate, and they are a safer farm product for consumers; hence, biofertilizers remains a better alternative for producing safer crops and enhancing global food security. In recent years, the plant nutrient gap between removal and supply through chemical fertilizer was over 10 million tons. Over-dependence on chemical fertilizers, in terms of both cost and environmental impact, is not a viable strategy in the long run due to the costs involved in setting up fertilizer plants and maintaining production, both in terms of domestic resources and foreign exchange. Biofertilizers are products that, once adequate information is available to producers and farmers, are likely to be commercially promising in the long run. The use of biofertilizers in the world will not only have an impact on the economic development of sustainable agriculture, but it will also contribute to a sustainable ecosystem and the overall wellbeing of humans.

## Figures and Tables

**Table 1:** Classification of biofertilizers and their mechanism of action.

Biofertilizers	Mechanism	Groups	Examples	References
Nitrogen-fixing	Increase the amount of N_2_ in the soil by fixing atmospheric nitrogen and making it available to plants.	Free-living, symbiotic, and associative symbiotic	*Aulosira bejerinkia*, *Nostoc*, *Klebsiella*, *Stigonema*, *Desulfovibrio*, *Azotobacter*, *Anabaena*, *Clostridium*, *Rhodospirillum*, and *Rhodopseudomonas* *Rhizobium*, *Frankia*, *Anabaena azollae*, and *Trichodesmium* *Azospirillum* spp., *Herbaspirillum* spp., *Alcaligenes*, *Enterobacter*, *Azoarcus* spp., and *Acetobacter diazotrophicus*	[17]
Phosphorus-mobilizing	Phosphorus is transferred from the soil to the root cortex. These are bio-fertilizers with a wide range of applications.	Mycorrhiza	*Arbuscular mycorrhiza*, *Acaulospora* spp., *Scutellospora* spp., *Glomus* spp., *Gigaspora* spp., and *Sclerocystis* spp.	[17]
Potassium solubilizing	Produce organic acids that degrade silicates and aid in the removal of metals to solubilize potassium (silicates) ions and make it available to plants.	Bacteria	*B. edaphicus*, *Arthrobacter* spp., *Bacillus*, *Mucilaginosus*, and *B. circulanscan*	[22]
		Fungi	*Aspergillus niger*	
Potassium mobilizing	They transfer potassium from the soil’s inaccessible forms.	Bacteria	*Bacillus* spp.	[23]
		Fungi	*Aspergillus niger*	
Phosphorus solubilizing	To dissolve bound phosphates, they secrete organic acids and lower soil pH by converting insoluble forms of P in the soil into soluble forms.	Bacteria, fungi	*Pseudomonas striata*, *Bacillus circulans*, *Bacillus subtilis*, *Penicilium* spp., *B. polymyxa*, *Agrobacterium*, *Microccocus*, *Flavobacterium*, *Aereobacterium. Aspergillus awamori*, *Penicillum* spp., and *Trichoderma* spp.	[17]
	Sulfur is oxidized to sulfate, which is the usable form for plants.	Sulfur-oxidizing	*Thiobacillus* spp.	[24]
Micronutrient	Protons, chelated ligands, acidification, and oxidoreductive systems can all be used to dissolve zinc.	Zinc-solubilizing	*Pseudomonas* spp., *Mycorhiza*, and *Bacillus* spp.	[25]
Plant growth-promoting	Produce hormones that encourage root growth, increase nutrient availability, and boost crop yields.	Plant growth-promoting rhizobacteria	*Agrobacterium*, *Pseudomonas fluorescens*, *Arthrobacter*, *Erwinia*, *Bacillus*, *Rhizobium*, *Pseudomonas* spp. *Enterobacter*, *Streptomyces*, and *Xanthomonas*	[17]

## Data Availability

Not applicable.
